# Oncologic Outcome and Immune Responses of Radiotherapy with Anti-PD-1 Treatment for Brain Metastases Regarding Timing and Benefiting Subgroups

**DOI:** 10.3390/cancers14051240

**Published:** 2022-02-27

**Authors:** Maike Trommer, Anne Adams, Eren Celik, Jiaqi Fan, Dominik Funken, Jan M. Herter, Philipp Linde, Janis Morgenthaler, Simone Wegen, Cornelia Mauch, Cindy Franklin, Norbert Galldiks, Jan-Michael Werner, Martin Kocher, Daniel Rueß, Maximilian Ruge, Anna-Katharina Meißner, Christian Baues, Simone Marnitz

**Affiliations:** 1 Department of Radiation Oncology, Cyberknife Center, Faculty of Medicine, University Hospital Cologne, University of Cologne, 50937 Cologne, Germany; eren.celik@uk-koeln.de (E.C.); jiaqi.fan@uk-koeln.de (J.F.); dominik.funken@uk-koeln.de (D.F.); jan.herter@uk-koeln.de (J.M.H.); philipp.linde@uk-koeln.de (P.L.); janis.morgenthaler@uk-koeln.de (J.M.); simone.wegen@uk-koeln.de (S.W.); christian.baues@uk-koeln.de (C.B.); simone.marnitz-schulze@uk-koeln.de (S.M.); 2 Center of Integrated Oncology (CIO), Universities of Aachen, Bonn, Cologne, and Düsseldorf, 50937 Cologne, Germany; cornelia.mauch@uk-koeln.de (C.M.); cindy.franklin@uk-koeln.de (C.F.); norbert.galldiks@uk-koeln.de (N.G.); jan-michael.werner@uk-koeln.de (J.-M.W.); martin.kocher@uk-koeln.de (M.K.); daniel.ruess@uk-koeln.de (D.R.); maximilian.ruge@uk-koeln.de (M.R.); anna-katharina.meissner@uk-koeln.de (A.-K.M.); 3 Center for Molecular Medicine Cologne, University of Cologne, 50937 Cologne, Germany; 4 Institute of Medical Statistics and Computational Biology, Faculty of Medicine, University Hospital Cologne, University of Cologne, 50937 Cologne, Germany; anne.adams@uni-koeln.de; 5 Department of Dermatology, Faculty of Medicine, University Hospital Cologne, University of Cologne, 50937 Cologne, Germany; 6 Department of Neurology, Faculty of Medicine, University Hospital Cologne, University of Cologne, 50937 Cologne, Germany; 7 Department of Neuroscience and Medicine (INM-3), Research Center Juelich, 52428 Juelich, Germany; 8 Department of Stereotactic and Functional Neurosurgery, Faculty of Medicine, University Hospital Cologne, University of Cologne, 50937 Cologne, Germany; 9 Department for General Neurosurgery, Centre of Neurosurgery, Faculty of Medicine, University Hospital Cologne, University of Cologne, 50937 Cologne, Germany

**Keywords:** radiotherapy, radioimmunotherapy, immune checkpoint inhibition, PD-1/PD-L1, brain metastases, malignant melanoma, stereotactic radiosurgery, whole brain radiotherapy, abscopal effects, pseudoprogression

## Abstract

**Simple Summary:**

Immune checkpoint inhibitors (ICIs) and radiotherapy (RT) are widely used for patients with brain metastasis (BM). To evaluate markers for treatment response and find a treatment concept which has the best outcome effects, we analyzed data of 93 patients with BM from different cancer types. Predictive markers for survival were good performance status, melanoma as cancer type, low metastasis volume, normal inflammatory blood parameters, and a stereotactic radiotherapy concept with high doses. We found that the best survival outcome can be achieved with the concurrent use of RT and ICI. Concurrent treatment was particularly beneficial in patients with low inflammatory status and more and larger metastases, and when high doses cannot be administered. In concurrently treated patients, therapeutic response was often delayed compared to sequential treatment. Specific immune responses such as pseudoprogression and abscopal effects were induced by concurrent treatment and associated with prolonged survival.

**Abstract:**

While immune checkpoint inhibitors (ICIs) in combination with radiotherapy (RT) are widely used for patients with brain metastasis (BM), markers that predict treatment response for combined RT and ICI (RT-ICI) and their optimal dosing and sequence for the best immunogenic effects are still under investigation. The aim of this study was to evaluate prognostic factors for therapeutic outcome and to compare effects of concurrent and non-concurrent RT-ICI. We retrospectively analyzed data of 93 patients with 319 BMs of different cancer types who received PD-1 inhibitors and RT at the University Hospital Cologne between September/2014 and November/2020. Primary study endpoints were overall survival (OS), progression-free survival (PFS), and local control (LC). We included 66.7% melanoma, 22.8% lung, and 5.5% other cancer types with a mean follow-up time of 23.8 months. Median OS time was 12.19 months. LC at 6 months was 95.3% (concurrent) vs. 69.2% (non-concurrent; *p* = 0.008). Univariate Cox regression analysis detected following prognostic factors for OS: neutrophil-to-lymphocyte ratio NLR favoring <3 (low; HR 2.037 (1.184–3.506), *p* = 0.010), lactate dehydrogenase (LDH) favoring ≤ULN (HR 1.853 (1.059–3.241), *p* = 0.031), absence of neurological symptoms (HR 2.114 (1.285–3.478), *p* = 0.003), RT concept favoring SRS (HR 1.985 (1.112–3.543), *p* = 0.019), RT dose favoring ≥60 Gy (HR 0.519 (0.309–0.871), *p* = 0.013), and prior anti-CTLA4 treatment (HR 0.498 (0.271–0.914), *p* = 0.024). Independent prognostic factors for OS were concurrent RT-ICI application (HR 0.539 (0.299–0.971), *p* = 0.024) with a median OS of 17.61 vs. 6.83 months (non-concurrent), ECOG performance status favoring 0 (HR 7.756 (1.253–6.061), *p* = 0.012), cancer type favoring melanoma (HR 0.516 (0.288–0.926), *p* = 0.026), BM volume (PTV) favoring ≤3 cm^3^ (HR 1.947 (1.007–3.763), *p* = 0.048). Subgroups with the following factors showed significantly longer OS when being treated concurrently: RT dose <60 Gy (*p* = 0.014), PTV > 3 cm^3^ (*p* = 0.007), other cancer types than melanoma (*p* = 0.006), anti-CTLA4-naïve patients (*p* < 0.001), low NLR (*p* = 0.039), steroid intake ≤4 mg (*p* = 0.042). Specific immune responses, such as abscopal effects (AbEs), pseudoprogression (PsP), or immune-related adverse events (IrAEs), occurred more frequently with concurrent RT-ICI and resulted in better OS. Other toxicities, including radionecrosis, were not statistically different in both groups. The concurrent application of RT and ICI, the ECOG-PS, cancer type, and PTV had an independently prognostic impact on OS. In concurrently treated patients, treatment response (LC) was delayed and specific immune responses (AbE, PsP, IrAE) occurred more frequently with longer OS rates. Our results suggest that concurrent RT-ICI application is more beneficial than sequential treatment in patients with low pretreatment inflammatory status, more and larger BMs, and with other cancer types than melanoma.

## 1. Introduction

Advanced-stage cancer patients develop brain metastases (BMs) in 20–40% of cases. BMs are most common in lung cancer, breast cancer, malignant melanoma, renal cancer, and carcinomas of the gastrointestinal tract. Current strategies for the management of BM include systemic therapy, surgery, and radiation therapy (RT), mostly applied as stereotactic radiosurgery (SRS) or whole brain radiotherapy (WBRT) [1,2]. Meanwhile, SRS is increasingly used in clinical routine since it shows comparable or even better outcomes associated with less toxicity compared to WBRT [3].

Immune checkpoint inhibitors (ICIs) act by releasing the inhibition of functional immune cells to restore their antitumor activity. Binding of programmed cell death ligand 1 (PD-L1), expressed by cancer cells, to its receptor programmed cell death protein 1 (PD-1) on T cells sends an inhibitory signal and leads to T cell dysfunction. Targeting the PD-1/PD-L1 checkpoint is an established treatment for many cancers. Another clinically used ICI targets the binding of anticytotoxic T-lymphocyte-associated protein 4 (CTLA4) to its ligands expressed by antigen-presenting cells [4].

ICI therapy has fundamentally changed oncologic treatment strategies even for difficult-to-treat advanced cancers such as malignant melanoma (MM), non-small cell lung cancer (NSCLC), renal cell carcinoma (RCC), squamous cell carcinoma of the head and neck (SCC), and liver and bladder cancer [5,6].

BM patients show increasingly longer survival times due to good response to therapies like ICIs with intracerebral effectiveness, which makes toxicity avoidance even more important. The highest intracerebral response rates are currently achieved by ICI combinations such as the combination of CTLA4 antibody with a PD-1 inhibitor. Intracranial treatment benefit can reach up to 57% including a complete response rate of 25% after 6 months in melanoma patients with BM while this is accompanied by a high number of immune-related adverse events (IrAEs) [7,8,9]. The combination of ICI with local RT might be one promising approach that enhances antitumor immune responses of ICI in BM patients while sparing toxicity as summarized in a recent review by Su et al. for BM in solid tumors including melanoma, RCC, and NSCLC with intracranial control rates of up to 100% after one year [10].

RT is known to induce systemic reactions by the modulation of the tumor and its microenvironment, increasing antigen presentation and recognition, leading to an improved antitumor immune response in various cancer types [10,11,12]. Clinical results of this combination approach support the preclinical rationales: In the treatment of NSCLC as well as esophageal cancer, ICI consolidation after RT showed better oncologic outcomes compared to placebo [13,14,15]. Additionally, in the treatment strategies of BM, preclinical and clinical trials favor the combination concept of RT and ICI treatment, especially when simultaneously applied [16,17].

Synergistic effects include abscopal effects (AbEs), the shrinkage of non-irradiated lesions as a sign of a systemic effect of RT [18,19,20], and the occurrence of pseudoprogression (PsP), the transient increase in contrast-enhancing lesions, which is a known imaging observation, e.g., after SRS for BM [21,22].

Prognostic factors that would allow a better prediction of treatment response and long-term survival with combined RT and ICI (RT-ICI) to BM are still under investigation. The optimal sequence of application is also still a matter of discussion.

In this study, we investigated prognostic factors for therapeutic outcome in patients with BM of different cancer types treated with RT-ICI and the effects of concurrent and non-concurrent application in different subgroups. Besides established risk factors associated with shortened survival, such as low performance status and presence of extracranial disease, we examined other covariates potentially affecting RT-ICI treatment outcome, such as different RT concepts, volume, and dosage, and factors associated with inflammation [23,24].

Furthermore, we analyzed toxicities including radionecrosis, and the impact of distinctive reactions of the immune system such as AbE, PsP, and IrAE on patient outcome.

## 2. Materials and Methods

### 2.1. Patients and Treatment

Patients with brain metastases (BMs) receiving the PD-1 inhibitors pembrolizumab or nivolumab at the University Hospital of Cologne between September/2014 and November/2020 were identified from electronic patient files. Ninety-three patients received additional RT to the brain and were included in this analysis. This research has been approved by the Ethics Committee of the University of Cologne, Faculty of Medicine (reference: 19–1160). We retrospectively analyzed patient, disease, and treatment characteristics, treatment outcome such as overall survival (OS), progression-free survival (PFS), and local control (LC), covariates with a possible impact on treatment outcome such as Eastern Cooperative Oncology Group Performance Status (ECOG-PS), body mass index (BMI), presence of extracranial disease, which was defined as manifestation of metastatic disease outside the brain, high tumor burden, number of BMs, total mean planned target volume (PTV) of all irradiated BMs, PD-L1 status, neutrophil-to-lymphocyte ratio (NLR) (calculated by dividing the neutrophil count by the lymphocyte count), neurological symptoms, biologically effective dose (BED), RT and ICI timing, dexamethasone intake, prior systemic treatment, as well as treatment-related toxicities. All measurements were taken at the start of RT unless otherwise stated.

Patients received RT and anti-PD-1 therapy either concurrently (start of RT and ICI within one month) or non-concurrently with at least a one-month interval between both therapies.

In case patients received more than one RT in their medical history, we referred to the RT given closest to the ICI schedule. In case patients received more than one regimen of PD-1 inhibitors in their medical history, we referred to the ICI treatment that was closest to the respective RT to the brain.

We included any RT concept regardless of the fractionation scheme and RT dose. In order to better compare total doses, we performed isoeffective dose calculations and obtained the BED for each RT concept. The formula for RT dose intensity assessment (BED modeling) was adapted from Fowler et al. [25]. The total PTV was calculated by summing all separate PTVs of each individual RT plan.

ICIs were intravenously applied. Pembrolizumab was administered at a dose of 2 mg/kg every three weeks and nivolumab at a dose of 3 mg/kg every two weeks.

### 2.2. Outcome Evaluation

OS and PFS were defined as the time from start of the respective RT to death from any cause (OS), systemic or cerebral progression, or last visit. We set the PFS event date as the radiological image with progression taken before change in treatment or disease-related death. LC was defined as the absence of any activity in intracranial disease (new BM or progression of existing lesions). Mixed response was not considered as local control. Overall response rate was assessed as complete response (CR), partial response (PR), stable disease (SD), or progressive disease (PD); accordingly, progression rate was assessed as cerebral progression, systemic progression, overall progression (cerebral and systemic), and no progression. Radiological outcome and treatment-related changes such as radionecrosis and pseudoprogression (PsP) were measured using magnetic resonance imaging (MRI) according to Response Evaluation Criteria in Solid Tumors (RECIST) version 1.1 and immune-related RECIST (iRECIST). In case of uncertainty, an additional *O*-(2-[^18^F]fluoroethyl)-L-tyrosine positron emission tomography (FET PET) was considered.

Abscopal effects (AbEs) were defined as regression of lesions outside the RT field, and pseudoprogression of irradiated lesions was characterized by a transient increase in contrast-enhancing lesions after RT, mimicking tumor progression. We classified a lesion as pseudoprogressive if it increased in the first follow-up MRI at 3 months and decreased or stabilized in follow-up imaging at 6 months without treatment change.

For outcome evaluation, we report medians with 95% CI in parentheses when possible. If the survival rate does not fall to 50% or below, we report the mean values. Differences in numbers of patients per group in tables, figures, and text are due to missing events.

### 2.3. Toxicity Evaluation

Adverse events (AEs) were analyzed according to the Common Terminology Criteria for Adverse Events (CTCAE) v5.0.

We evaluated the following immune-related adverse events (IrAEs): Pneumonitis, colitis including diarrhea, hepatitis, hyper- and hypothyroidism, pancreatitis, arthritis, myositis, and skin reactions such as pruritus (without distinction between early and late onset), and the following acute CNS toxicities: Nausea, vertigo, cephalgia, and/or visual disorders occurring within the first 3 months after RT. In addition, the frequency of patients with radionecrosis was assessed.

### 2.4. Statistical Analysis

All statistical analyses were performed using SPSS v. 28 (IBM Corp, Armonk, NY, USA). Patient and treatment characteristics, as well as AEs, were compared by the Kruskal–Wallis test for continuous variables and Pearson’s chi-square test for categorical variables where appropriate. OS, PFS, and local control were estimated by the Kaplan–Meier method and curve comparisons were performed using the log rank test. Non-event cases were censored in outcome analyses. We performed univariate and multivariate Cox proportional hazards regression analyses to evaluate the effect of baseline patient, disease, and treatment characteristics, as well as predefined covariates on OS. The following factors were included in the multivariate Cox regression analysis: ECOG, cancer type, RT concept, BED, RT and ICI timing, and prior anti-CTLA4 treatment. Due to a high number of missing values for NLR and lactate dehydrogenase (LDH), these covariates could not be included in the multivariate analysis. Patients with missing values were excluded from the respective analysis. In any case, *p*-values < 0.05 were considered significant and refer to two-sided tests.

## 3. Results

### 3.1. Patient, Lesion, and Treatment Characteristics

**Figure 1:**
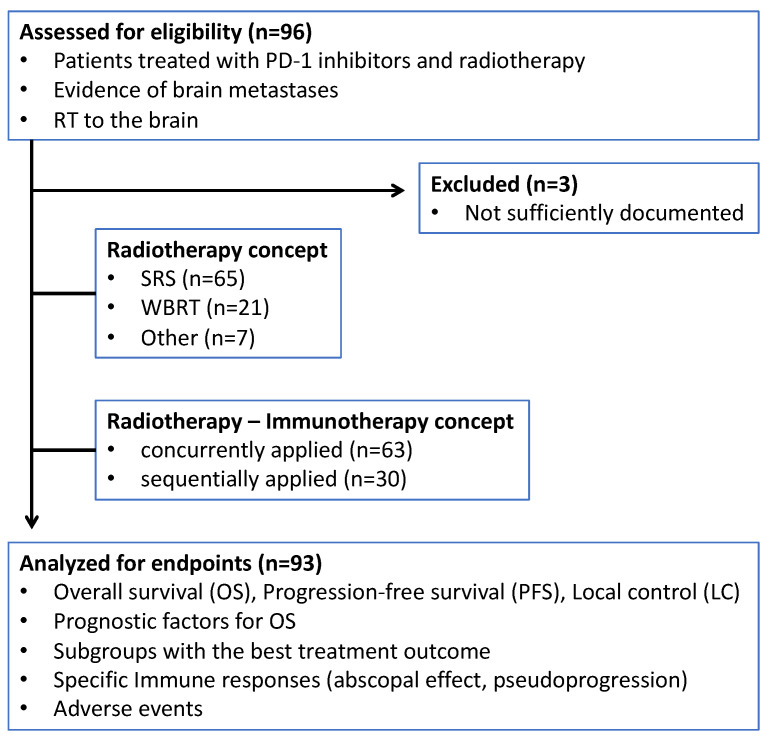
Study design. PD-1 = programmed cell death protein 1; RT = radiotherapy; SRS = stereotactic radiosurgery; WBRT = whole brain radiation therapy.

Altogether, 93 patients with 319 BMs met our inclusion criteria and were eligible for analysis (Figure 1). Fourteen of these had 10 or more BMs. In addition to RT, 67.7% of patients received pembrolizumab (*n* = 63) and 32.3% nivolumab (*n* = 30). Baseline and treatment characteristics for the entire cohort are demonstrated in Table 1.

Of our analyzed patients, 40.9% were female and 59.1% male with a mean age of 62.1 ± 13.2 years. Eastern Cooperative Oncology Group (ECOG) performance score was 0–1 in 77.6% of the cases despite almost half of the patients presenting additional extracranial disease (47.1%). Patients suffered from malignant melanoma (MM) in 70.7% of the cases. Other included malignancies were NSCLC (22.8%), RCC (1.1%), NHL (2.2%), and other (3.3%: SCLC, thymus carcinoma, breast cancer).

When compared to patients with MM, patients with other cancer types showed some significantly different baseline characteristics (see Appendix A Table A3). Significant differences were found in age with MM patients being older (*p* = 0.049), ECOG-PS with higher scores in patients with other cancers (*p* = 0.049), PTV with lower volumes in MM patients (*p* = 0.036), BED with higher doses in MM patients (*p* = 0.004), more prior systemic treatment in patients with other cancers (*p* < 0.001), and prior ipilimumab therapy only in MM patients (*p* < 0.001).

Regarding the entire cohort, LDH was mostly within the upper limit of normal (ULN) (55.9%) and NLR was mostly high (≥3) in 55.1% of the cases.

At the start of RT, 38.5% of the patients had >2 BM and 43.2% had neurological symptoms. Mean PTV was 277.5 (±452.9) cm^3^. Of the patients, 69.9% received SRS, 22.6% WBRT, and 7.5% other RT concepts (postoperative hypofractionated RT, postoperative conventionally fractionated RT, brachytherapy).

Mean BED was 55.7 ± 10.1 Gy. During the treatment, 19.8% of patients received therapeutic (>4 mg) and 68.6% prophylactic dexamethasone. Irrespective of timing and location, 64.8% had been irradiated more than once in their lifetime. Most patients (68.5%) had received prior systemic treatment.

Sixty-three (67.7%) patients were treated with RT and ICI concurrently, 30 (32.3%) received both therapies non-concurrently and, of these, 19 patients received RT before ICI and 11 after the last ICI application. The mean duration of ICI administration was 22.2 ± 22.8 weeks.

Except for the total PTV of BM, which was larger in the concurrently treated group (*p* = 0.046), and LDH, which was more often elevated in the non-concurrently treated group (*p* = 0.006), the assessed characteristics did not differ significantly between the two treatment groups (see Table 2).

### 3.2. Outcome Evaluation and Treatment Response

For detailed follow-up and outcome data for the entire cohort of RT-ICI patients, see Table 3.

Mean follow-up time for the entire cohort was 23.8 ± 24.3 months. By the time of database closure, 23 patients (25.8%) were alive.

The median OS time was 12.19 (4.36–20.02) months with a 12-month OS rate of 50.7%.

The median PFS time of the entire cohort was 4.70 (2.53–6.86) months.

LC after 3 months was 69.3% and after 6 months 89.3%.

Regarding overall response to treatment, 40.2% of all RT-ICI patients showed a clinical benefit (CR + PR + SD) and 59.8% PD.

An overall progression (cerebral + systemic) was detectable in 42.9% of all patients, only cerebral progression in 16.5%, and only systemic progression in 14.3%. By the time of data lock, 26.4% of the patients had no progression. Cerebral response rate was 42.9%.

#### 3.2.1. Impact on Survival of the Entire RT-ICI Cohort

**Figure A1:**
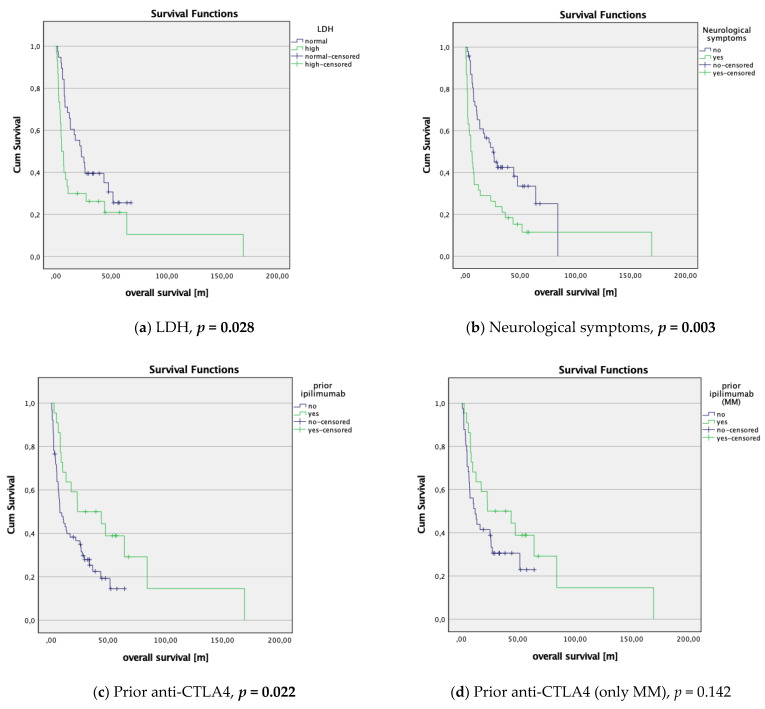

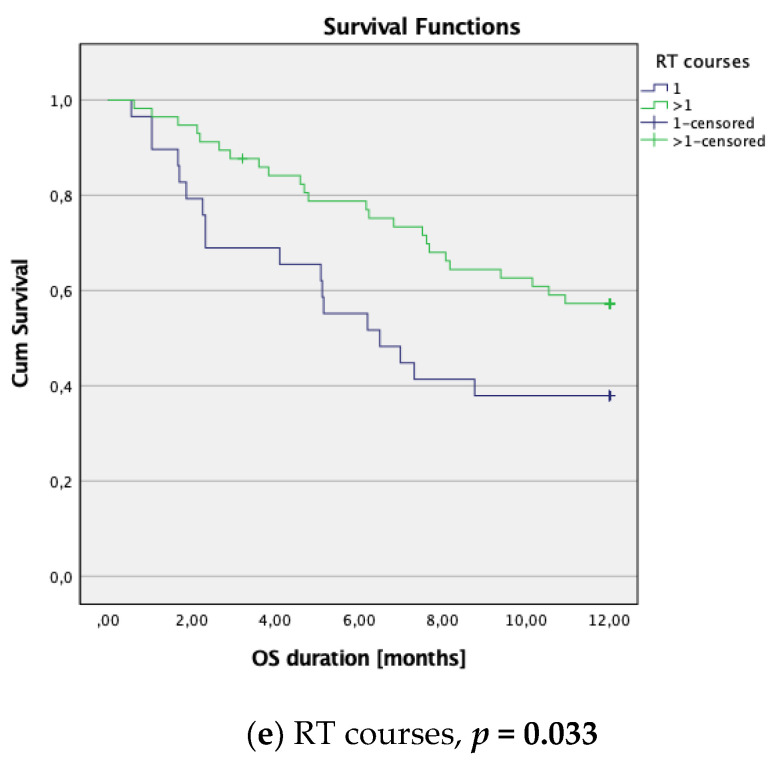
Kaplan–Meier curves for OS of the entire cohort RT-ICI. Kaplan–Meier curve comparisons were calculated using log rank test. Non-event cases are censored. (**a**) lactate dehydrogenase (LDH), (**b**) neurological symptoms, (**c**) prior anticytotoxic T-lymphocyte-associated protein 4 (anti-CTLA4), (**d**) prior anti-CTLA4 excluding cancer types other than malignant melanoma (MM), (**e**) applied RT courses at 12-month OS, cutoff 1. OS = overall survival, RT = radiotherapy, ICI = immune checkpoint inhibitor.

In the univariate Cox proportional hazard regression analysis, we found the following significant prognostic factors for OS: ECOG-PS ≥ 2 vs. 0 (HR 2.532; 95% CI 1.228–5.222, *p* = 0.012); cancer type favoring MM compared to other cancer types (HR 0.457, 95% CI 0.267–0.782, *p* = 0.004); NLR favoring < 3 (low; HR 2.037, 95% CI 1.184–3.506, *p* = 0.010); LDH favoring ≤ ULN (HR 1.853, 95% CI 1.059–3.241, *p* = 0.031); PTV favoring ≤ 3 cm^3^ (HR 2.213, 95% CI 1.305–3.754, *p* = 0.003); the absence of neurological symptoms at RT start (HR 2.114, 95% CI 1.285–3.478, *p* = 0.003); RT concept favoring SRS compared to WBRT (HR 1.985, 95% CI 1.112–3.543, *p* = 0.019); BED favoring ≥60 Gy (HR 0.519, 95% CI 0.309–0.871, *p* = 0.013); timing of the RT-ICI application favoring concurrent treatment after 12 months of follow-up (HR 0.527, 95% CI 2.86–0.973, *p* = 0.041); RT timing favoring concurrent treatment compared to RT after ICI (HR 3.971, 95% CI 1.839–7.814, *p* < 0.001; overall log rank: *p* < 0.001); and prior anti-CTLA4 treatment favoring its administration (HR 0.498, 95% CI 0.271–0.914, *p* = 0.024). Regarding the latter, there only remained a trend when considering only MM patients (see Appendix A Figure A1c,d).

For PFS we found the following significant prognostic factors in the univariate Cox regression analysis: Number of BMs favoring ≤2 (HR 1.616, 95% CI 1.004–2.599, *p* = 0.048); PTV favoring ≤3 cm^3^ (HR 1.819, 95% CI 1.124–2.943, *p* = 0.015); RT concept favoring SRS compared to WBRT (HR 1.828, 95% CI 1.053–3.174, *p* = 0.032); BED favoring ≥60 Gy (HR 0.599, 95% CI 0.370–0.969, *p* = 0.037); RT courses in medical history favoring >1 (HR 0.620, 95% CI 0.386–0.995, *p* = 0.048); RT timing favoring concurrent compared to RT after ICI (HR 2.104, 95% CI 1.079–4.099, *p* = 0.029).

For the detailed univariate Cox regression analysis regarding OS and PFS, see Table 4. Additional covariates analyzed that did not reach statistical significance are listed in Table A1 and Table A2.

When adjusted for confounding factors using multivariate Cox proportional hazard regression analysis, we found the following independent prognostic factors for OS: ECOG favoring 0 vs. ≥2 (HR 7.756, 95% CI 1.253–6.061, *p* = 0.012), cancer type favoring MM (HR 0.516, 95% CI 0.288–0.926, *p* = 0.026), the timing of the application of RT and ICI favoring concurrent compared to non-concurrent (HR 0.539, 95% CI 0.299–0.971, *p* = 0.040), and PTV favoring ≤3 cm^3^ (HR 1.947, 95% CI 1.007–3.763, *p* = 0.048).

In an additionally performed multivariate Cox regression analysis including LDH (favoring normal, *p* = 0.008) or NLR (favoring low, *p* = 0.025), these also showed a statistically significant prognostic impact on OS (data not shown). These analyses were not included here because of too many missings for both variables.

For the detailed multivariate Cox regression analysis, see Table 5.

**Figure 2:**
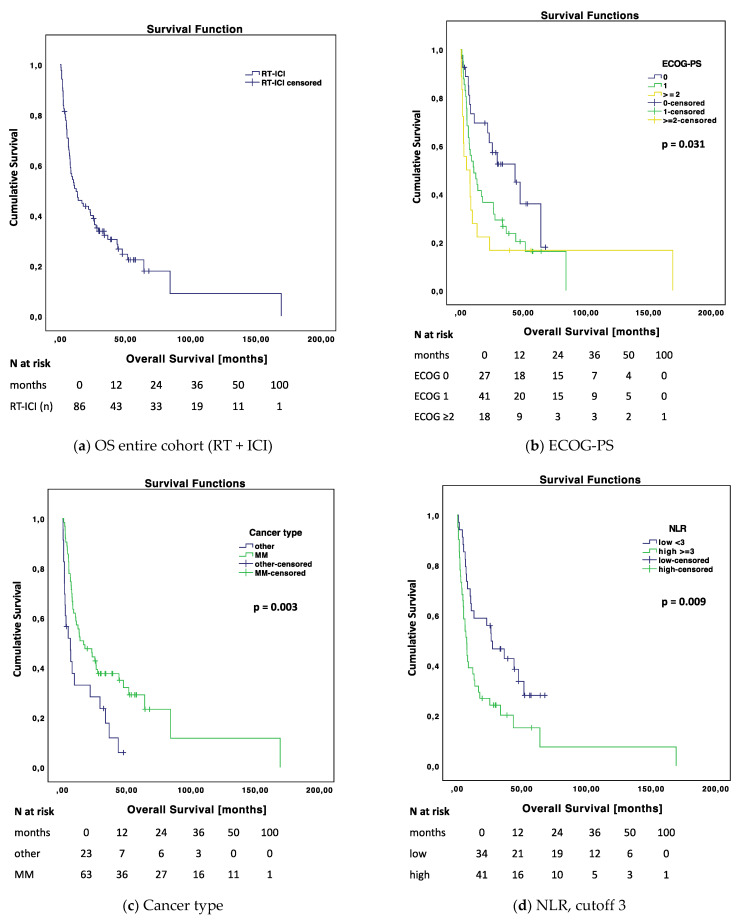

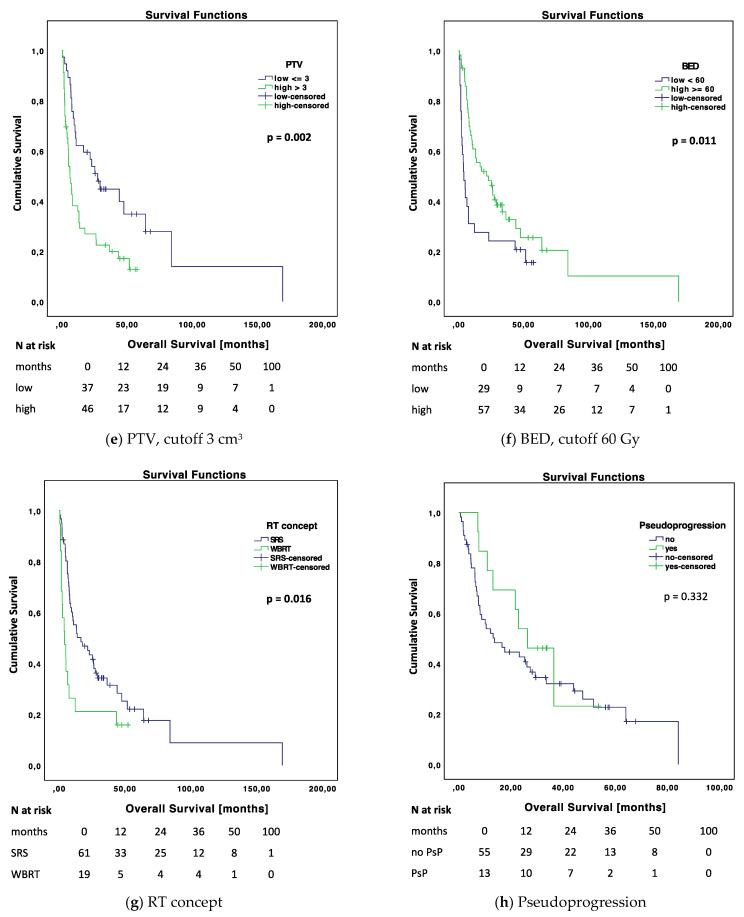

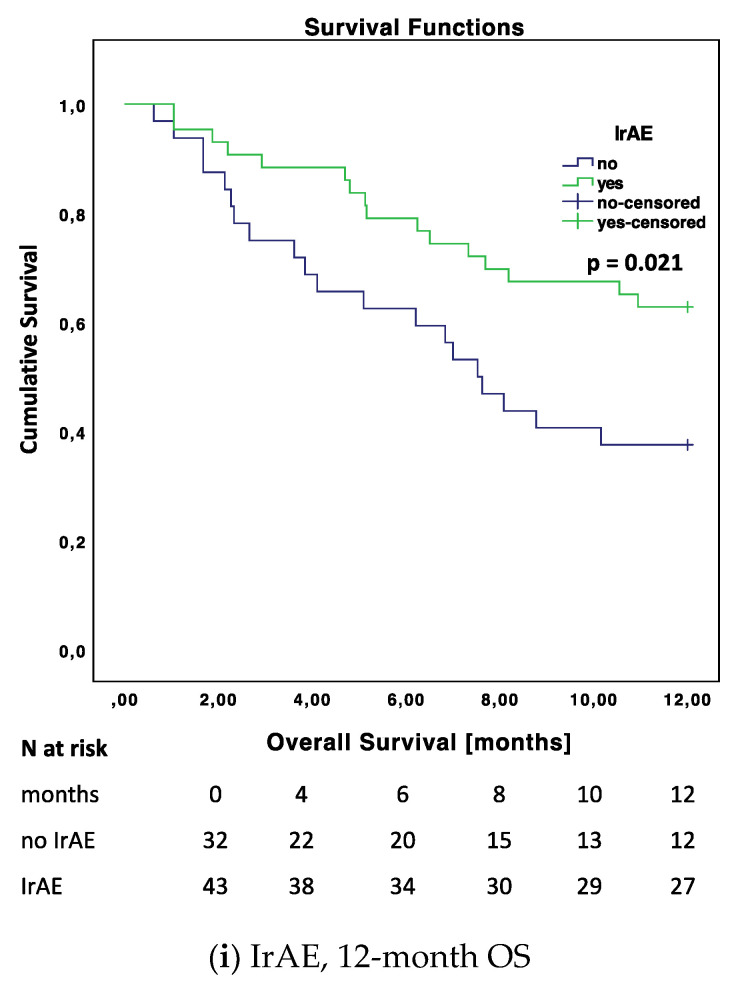
Kaplan–Meier curves for OS of the entire RT-ICI cohort regarding different covariates. Kaplan–Meier curve comparisons were calculated using log rank test. Non-event cases are censored. (**a**) Entire cohort RT-ICI; considering different covariates: (**b**) Eastern Cooperative Oncology Group Performance Status (ECOG-PS), (**c**) cancer type (MM = malignant melanoma), (**d**) NLR (neutrophil-to-lymphocyte ratio), cutoff 3, (**e**) planning target volume (PTV), cutoff 3 cm^3^, (**f**) biologically effective dose (BED), cutoff 60 Gy, (**g**) radiotherapy (RT) concept, SRS = stereotactic radiosurgery; WBRT = whole brain radiation therapy, (**h**) pseudoprogression, (**i**) immune-related adverse events (IrAEs) at 12-month OS.

Selected OS rates are shown in Appendix A Table A4 and corresponding selected Kaplan–Meier curves in Figure 2 and Appendix A Figure A1.

#### 3.2.2. Timing of RT-ICI Application with Regard to Different Subgroups

For detailed follow-up and outcome data regarding the timing of RT-ICI application, see Table 6.

Significant differences between both treatment groups were detected for LC, progression rate, and the occurrence of AbEs.

LC after 3 months was 81.8% in the non-concurrent RT-ICI group. The concurrent RT-ICI group showed an LC after 3 months of 64.2% with mixed lesion response in 13.8% of the cases. Non-concurrently treated patients showed an LC after 6 months of 69.2% and the concurrently treated patients of 95.3% with mixed lesion response in 4.1% of the cases. The difference in LC after 6 months was statistically significant (*p* = 0.008).

The progression rate of the non-concurrently treated RT-ICI group demonstrated overall progression in 56.7%, cerebral progression in 10%, and systemic progression in 23.3% The concurrently treated group showed overall progression in 36.1% of the cases, cerebral in 16.4%, and systemic in 9.8%. Of the concurrently treated patients, 37.7% showed no progression. The differences in progression rate were statistically significant (*p* = 0.015).

AbEs were rare and occurred only in the group with concurrent RT-ICI (*p* = 0.039).

PsP also occurred more frequently in the group with concurrent RT-ICI (*n* = 12; 22.6%) than in the group with non-concurrent RT-ICI (*n* = 1; 5%; *p* = 0.079).

**Figure 3:**
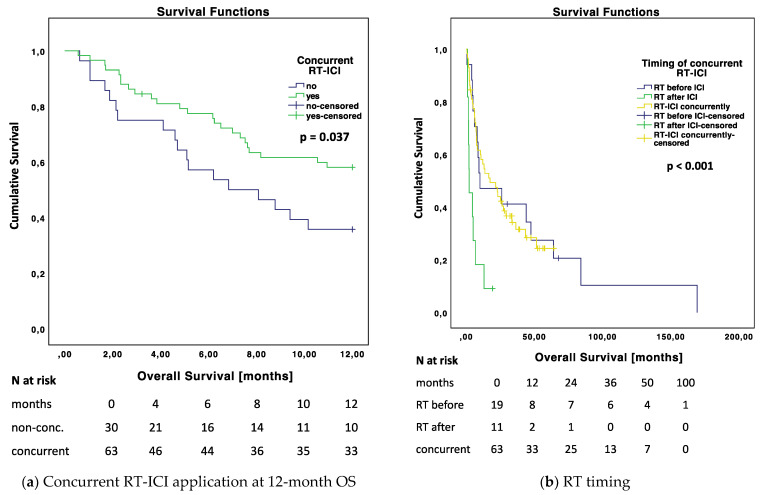

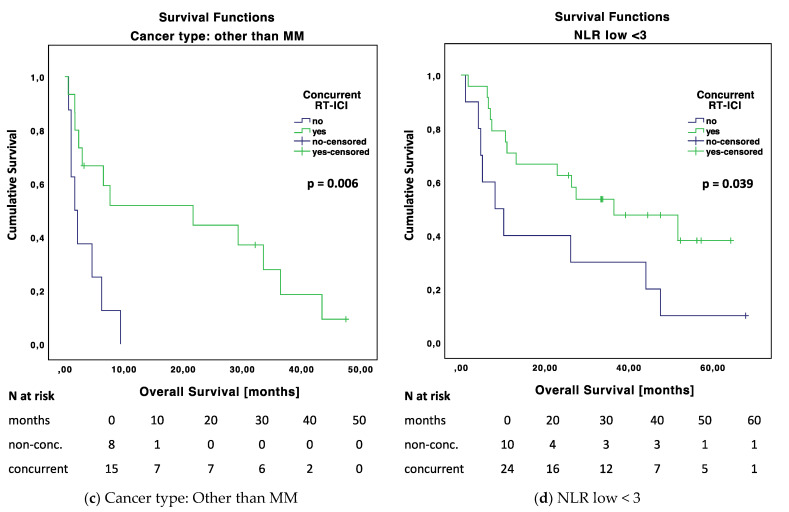

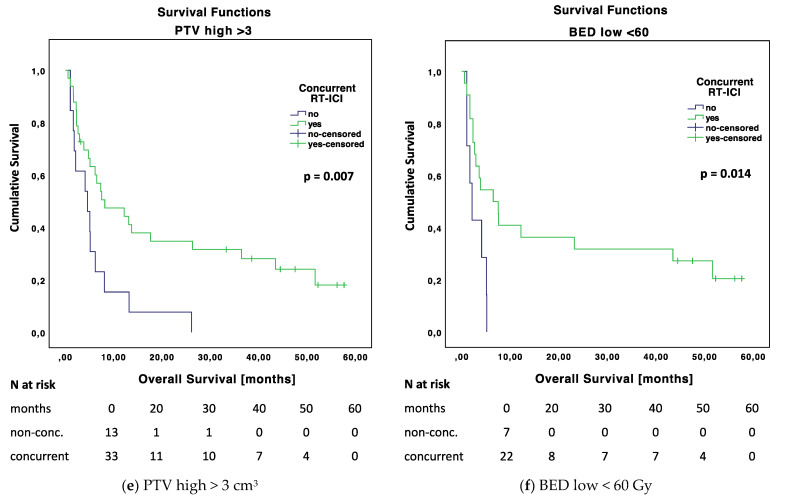

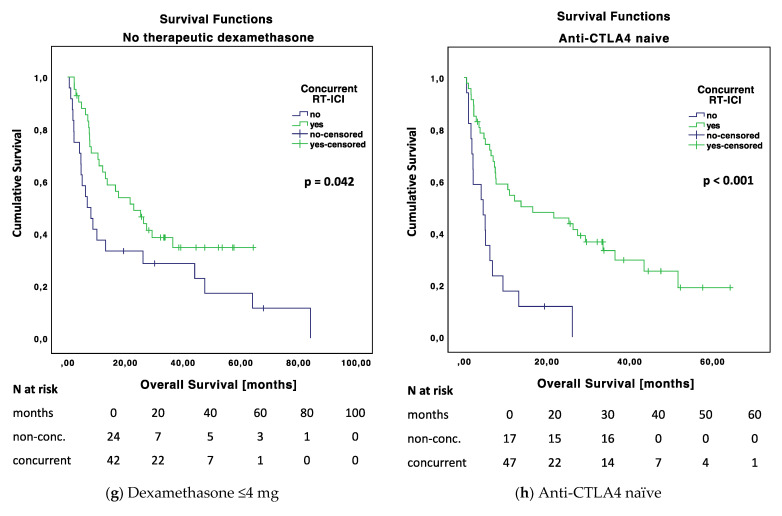

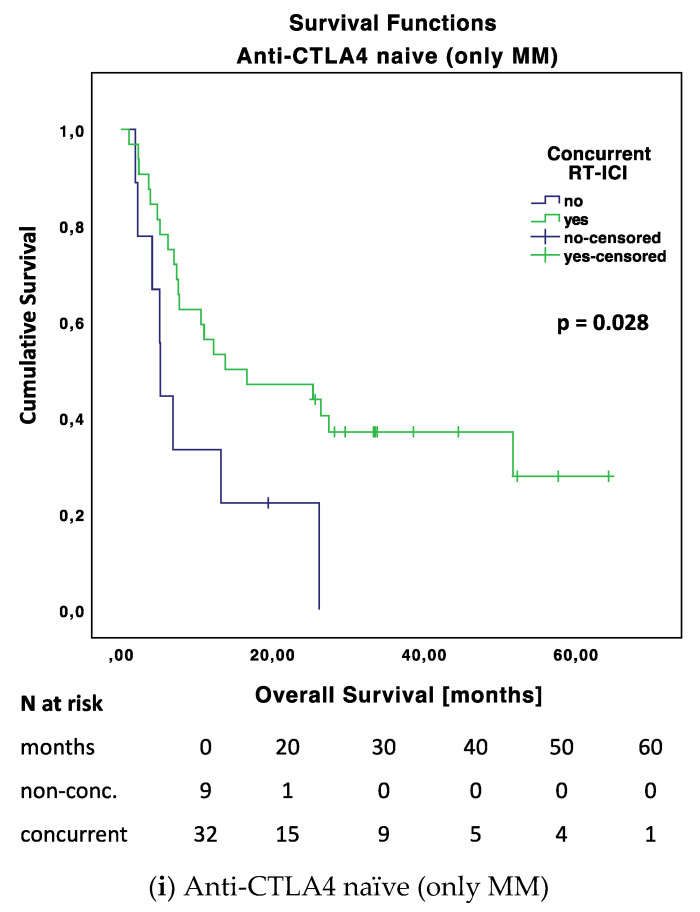
Kaplan–Meier curves for OS comparing concurrent RT-ICI and non-concurrent RT-ICI in different subgroups: (**a**) Entire cohort and (**b**) regarding timing; considering the different subgroups: (**c**) Cancer type: Other than malignant melanoma (MM), (**d**) neutrophil-to-lymphocyte ratio (NLR) low <3, (**e**) planning target volume (PTV) high >3 cm^3^, (**f**) biologically effective dose (BED) low <60 Gy, (**g**) dexamethasone intake ≤4 mg, (**h**) no prior anticytotoxic T-lymphocyte-associated protein 4 (anti-CTLA4), (**i**) no prior anti-CTLA4 in MM patients. OS = overall survival, RT = radiotherapy, ICI = immune checkpoint inhibitor.

**Figure A2:**
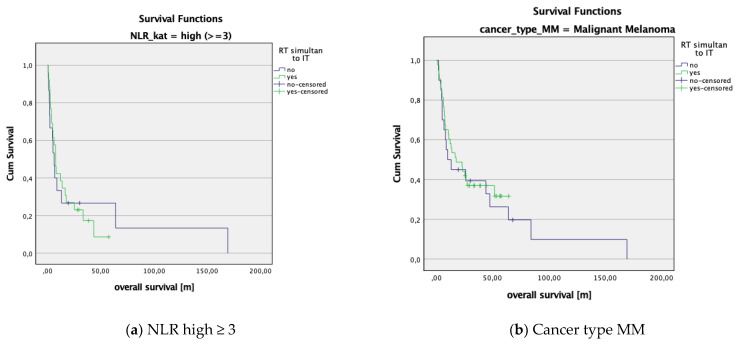

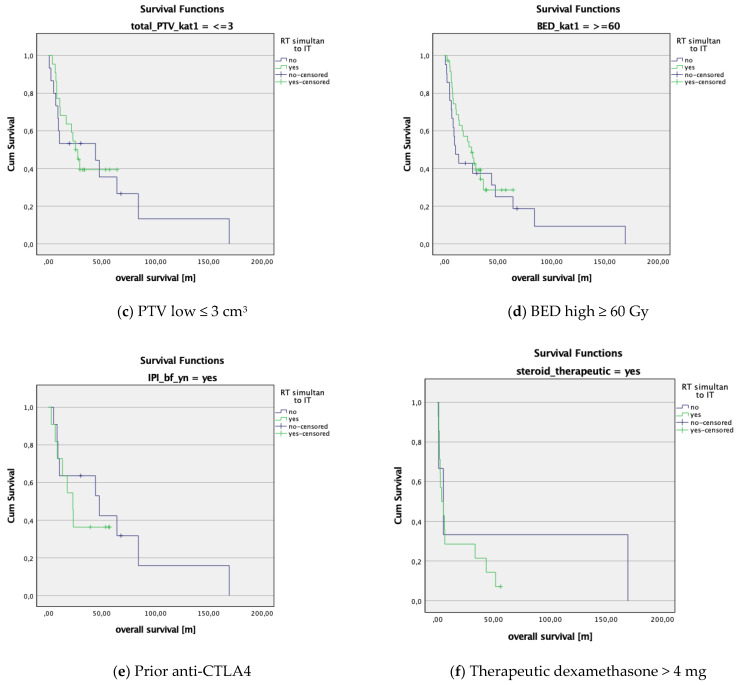
“Opposing” Kaplan–Meier curves for OS comparing concurrent RT-ICI and non-concurrent RT-ICI in different subgroups: (**a**) neutrophil-to-lymphocyte ratio (NLR) high ≥3, (**b**) cancer type malignant melanoma (MM), (**c**) planning target volume (PTV) low ≤3 cm^3^, (**d**) biologically effective dose (BED) high ≥60 Gy, (**e**) prior anticytotoxic T-lymphocyte-associated protein 4 (anti-CTLA4), (**f**) dexamethasone intake >4 mg. OS = overall survival, RT = radiotherapy, ICI = immune checkpoint inhibitor.

With regard to the timing of RT-ICI application, we compared different subgroups in terms of OS. Selected Kaplan–Meier OS curves with respect to RT-ICI timing and different subgroups are shown in Figure 3, the corresponding Kaplan–Meier curves are shown in Appendix A Figure A2.

When comparing concurrent and non-concurrent application, we found significant differences in the subgroup of patients with cancer types other than melanoma (*p* = 0.006); when PTV was >3 cm^3^ (*p* = 0.007); in the group of patients with low NLR (*p* = 0.039); with BED <60 Gy (*p* = 0.014); with dexamethasone intake of ≤4 mg (*p* = 0.042); and in the subgroup of anti-CTLA4-naïve patients (*p* < 0.001), also when considering only melanoma patients (*p* = 0.028).

### 3.3. Toxicity Evaluation

Adverse events (AEs) were recorded in 74.1% of all patients. Most AEs were CTCAE grade 1 or 2 (71.7%). We detected more IrAEs (61.5% vs. 46.2%), more acute CNS toxicities (42% vs. 22.2%), and more radionecrosis (11.7% vs. 3.4%) in the concurrent RT-ICI group, but these were not statistically significant. Adverse events are shown in detail in Appendix A Table A5.

## 4. Discussion

In this study we evaluated the effects of a combination treatment with RT to the brain and anti-PD-1 inhibitors (RT-ICI) applied concurrently or non-concurrently in a cohort of 93 patients with 319 brain metastases from different cancer types. To predict treatment response and long-term survival, we aimed at identifying prognostic factors for oncologic outcome parameters such as OS, PFS, and LC. Having proved that concurrent use of RT-ICI is an independent prognostic marker for OS, we consequently defined subgroups that benefit most in terms of the concurrent application of both treatments. In addition, we analyzed response rates and distinctive immune reactions to this immunogenic treatment combination, such as AbEs and PsP, as well as toxicities focusing on IrAEs, CNS, and radionecrosis.

### 4.1. Impact on Survival, Response Rate, and Oncologic Outcome

Most of the patients included in this study were diagnosed with cerebral metastasized MM and received SRS to two or fewer BMs. In univariate and multivariate Cox regression analysis performed in our study, the cancer type MM appeared to be an independent significant prognostic marker for OS (see Table 4 and Table 5). Regarding the differences in patient, lesion, and treatment characteristics of MM vs. other cancer types (see Appendix A Table A3), it is noticeable that the MM subgroup contains patients with better preconditions.

The fact that melanoma patients in our patient cohort showed a significantly longer OS is in line with current observations that those patients have increasingly longer survival times, as a good response to newer therapies such as ICI ensures a significantly longer disease course, which in many cases corresponds to the course of a chronic disease [26]. This makes toxicity avoidance more and more important.

Cerebral response rates have been shown to be better with combined PD-1 and CTLA4 inhibitors than with monotherapy. However, this is at the cost of an increased toxicity profile [7].

Whether and how BM should additionally be locally treated depends on size, number, and symptoms. Surgery, SRS, or hypofractionated radiotherapy and WBRT or a combination of these approaches are possible. WBRT has been frequently replaced by SRS [27]. Large randomized trials show marginally better CNS control rates after WBRT, but also often worsened neurocognition and quality of life without significant differences in OS [3]. WBRT is usually recommended for patients with extensive symptomatic BM and an expected lifetime of more than 3 months [28]. This is how WBRT was used in our study and how it is reflected in the data collected. This might be one reason why SRS showed a significantly better OS (*p* = 0.016) and was significantly associated with improved OS and PFS in the univariate analysis, however, not in the multivariate analysis.

In the past, SRS has been restricted to few metastases of ≤ 3 cm, but recent data have shown that stereotactic RT can also be used as single or fractionated treatment in the management of larger and more BMs [29,30]. Local control has been shown to be equivalent when >5 BMs are treated with SRS, with equal outcomes and no more adverse events [31,32]. Local control after SRS for BM, depending on underlying disease, volume of lesion, and dose is 73–94% with a low toxicity profile [31]. There are a number of studies showing that combination therapy of ipilimumab (CTLA4 inhibitor) with nivolumab (PD-1 inhibitor) has excellent efficacy in patients with MM. When including patients with BM, smaller studies usually exclude patients with large and symptomatic lesions and previous local therapy [7,9,16].

The CheckMate-204 trial enrolled 101 neurologically asymptomatic patients with MM in good general health (ECOG 0–1) with at least one BM ≤ 3 cm in diameter, of whom 57% showed intracranial treatment benefit: 25% responded with CR and 30% with PR after 6 months. Therapy-related CNS toxicity was seen in 36% with even 19.4% CTCAE grade 3 and 4 adverse events. In an update of the study, the authors reported the 20.6 months of follow-up, with a stable intracranial treatment benefit in asymptomatic patients and any response in four out of 18 (22.2%) symptomatic patients [9].

Even though there are more and more studies with systemic therapy for patients with BM alone, these must compete with the excellent local control rates and the low toxicity profile of SRS to replace the standard of local therapy.

In this analysis, we included patients with any ECOG-PS, and among these 22.5% had a score ≥2. A higher ECOG performance score was associated with significantly shorter OS (see Table 4 and Table 5). Presenting >2 BMs in 38.5% of the cases, almost half of our patient collective showed neurological symptoms, which also proved to have a significant impact on OS. Despite this real-world collective with bad prognosis, we observed an overall clinical benefit (intra- and extracranial) of 40.2% after 23.8 months of follow-up, showing intracerebral response in 42.9% of the cases. Local control after 6 months was 89.3% for all patients regardless of RT type with acute CNS toxicity of 35.1% with no CTCAE grade 4 and 11.1% grade 3 events.

The number of BMs had a significant impact on PFS and the volume of all treated metastases (PTV) was an independent prognostic factor for OS. Regarding different PTV sizes, we found a significant difference between 1–3 cm^3^ and >3 cm^3^ (*p* = 0.002) or ≤3 cm^3^ vs. >10 cm^3^. According to this, we set a cutoff for PTV at 3 cm^3^. Patients with a low PTV had a significantly longer OS.

The number and volume of BMs plays a distinct role regarding the OS. BMs therefore should be treated as early as possible.

The RT concept also had a significant impact on OS and PFS. Patients receiving SRS additionally to ICI showed a significantly longer OS than those with WBRT. These results are in accordance with the indication for WBRT for symptomatic patients with multiple BMs and a poor prognosis. Regarding the applied RT dose, patients being irradiated with ≥60 Gy had a significantly longer OS.

In terms of additional treatment, we analyzed prior RT, RT courses, and prior systemic treatment. A number of RT courses > 1 had a significant impact on PFS, and when analyzing the 12-month follow-up, also on OS, see Figure A1e. Arguably, the need for a certain “lifetime” RT dose may be hypothesized that provides the highest immunogenic benefit because more diverse neoantigens are released and the immune system is sensitized to boost immunogenic effects [33].

### 4.2. Combination of RT-ICI and Timing

Concurrent application of RT-ICI proved to be an independent prognostic marker for OS in our study. Regarding the 12-month OS, patients with concurrent RT-ICI lived significantly longer. Concurrent application showed the best OS rates (12 months: 58.1% (concurrent), 47.1% (before ICI) and 18.2% (after ICI)), however, only 11 patients (11.8%) received RT after ICI.

Regarding LC, the non-concurrent RT-ICI group showed a better LC after 3 months while the concurrent RT-ICI group demonstrated a lot of mixed responses. After 6 months, however, the concurrent RT-ICI group showed a significantly better LC (95.3% vs. 69.2%, *p* = 0.008) and had more patients with smaller or stable lesions (3 months: 51.7%, 6 months: 75.5%). This might be due to PsP or delayed reactions of the immune system with simultaneous RT-ICI application, which is discussed further in Section 4.4 on distinctive reactions of the immune system to RT-ICI.

Overall, there are a number of rationales for combining ICI and RT, but there are few solid data available.

First, RT has been shown to induce all three types of immunogenic cell death. The activation of cell death is related to the presence of damage-associated molecular patterns (DAMPs) on the cell surface, which causes mobilization of immune cells and affects their function. By killing tumor cells, RT helps to release antigens and cytokines and upregulates MHC-I molecules, which triggers immune responses. ICIs are used to prevent these reactions from being triggered by the tumor itself.

Second, RT can induce the expression of PD-L1 on tumor cells. Tumors with low or negative PD-L1 status may become more sensitive to ICI this way.

Third, RT leads to increased invasion of immune cells in brain tumors, presumably by softening the blood–brain barrier. ICI may enhance the local effect of RT [10,11,12,34,35].

These rationales suggest that there may be a survival benefit for patients with any RT concept and ICI therapy, which can be explained by the increased synergistic immunogenic reactions due to the combination therapy. The right RT dosage, type of fractionation, and timing of application of both therapeutic modalities, however, remain unclear to date.

ICIs were actually first used in MM and NSCLC for cerebral metastases in combination with RT. Here, mostly retrospective studies are available. Prospective investigation in trials has only been conducted for a few years, so results are limited but increasing [36].

Several small studies and meta-analyses have shown that patients treated with ICI and concurrent SRS have better OS than patients treated with ICI and non-concurrent SRS [37,38,39].

A better survival probability in both asymptomatic and symptomatic patients after SRS or surgery was also observed in a retrospective study by Amaral et al. including 380 patients with melanoma brain metastasis (MBM) treated with PD-1+/-CTLA4 inhibitors. The positive effect was evident throughout with a trend toward local therapy upfront [16].

Opijnen et al. concluded in their review that the combination of RT with ICI can achieve better tumor control and longer survival in MBM patients, although the results of the 95 included studies are heterogeneous and, in some cases, contradictory. Timing appears to be an important factor, with the best results obtained when RT was delivered before or during ICI [17]

### 4.3. Subgroup Analyses Regarding Concurrent Application of RT-ICI

Considering the evidence that application of RT during ICI treatment leads to the best oncologic outcomes, we analyzed subgroups regarding the concurrent timing of RT-ICI application. Figure 3 shows the subgroups that were significantly associated with better OS when RT-ICI was applied concurrently.

We were able to show, in another study, that the subgroup of patients with advanced-stage cancers other than MM benefited more from the combination of RT-ICI compared to anti-PD-1 treatment alone [40]. This supports the results we found in this study, that those patients seem to particularly benefit from the concurrent RT-ICI combination. It is likely that melanoma patients respond more effectively to systemic treatment with ICI only and RT may not have such a strong additional effect on it as it does in other cancer types [40].

The NLR as a marker of systemic inflammation has long been discussed as a prognostic marker in different cancer types.

A high pretreatment NLR seems to be associated with poorer survival outcomes. Especially in patients receiving RT, having advanced-stage cancers, or MM, NLR demonstrates stronger associations with survival [41]. Many previous studies have examined the prognostic value of pretreatment NLR [42,43].

As our median NLR was 3.35, we set the cutoff for high NLR at ≥3, as defined in other studies [44,45]. A low NLR <3 proved to be a prognostic marker for OS in our analysis of the entire cohort. The subgroup analysis showed that patients with low NLR values have significantly prolonged OS when being treated concurrently with RT-ICI. This may provide further evidence to the suggestion that RT transforms “cold tumors” with low inflammatory status into “hot tumors”, leading to improved efficacy of ICI treatment [46].

Considering BM number, irradiated volume (PTV), delivered RT courses, and dose (BED), it appears that patients with a presumed worse prognosis are more likely to benefit from concurrent RT-ICI treatment, i.e., those with a high volume of >3 cm^3^ and a low applied RT dose of <60 Gy. After 12 months’ follow-up time, patients with >1 RT courses in their medical history, >2 BMs, and neurological symptoms also show statistically significantly longer OS with concurrent RT-ICI application. We found no difference for the RT concepts SRS or WBRT in the subgroup analysis. It must be assumed that the improved OS rates also depend on the prompt application of the RT. Consequently, patients with a higher “lifetime” RT dose, more and larger BMs, and neurological symptoms, in whom a sufficient or ablative dose cannot be administered, should especially be treated early and with RT-ICI concurrently rather than sequentially, to achieve better OS rates.

Anti-CTLA4-naïve patients showed in the subgroup analysis a significantly longer OS when being treated concurrently. With regard to the inflammatory status, it could be assumed that the stimulus for inflammation has already been set in CTLA4-pretreated patients, so the timing of RT-ICI application makes no difference in these patients [46]. Since patients with malignancies other than MM had no prior ipilimumab (see Appendix A Table A3), we censored the patients with other cancer types. In the subgroup of MM only, anti-CTLA4-naïve patients still had significantly longer OS rates (see Figure 3i). Prior anti-CTLA4 treatment is therefore an important selection criterion for RT-ICI. When there is an indication for RT-ICI in patients who are anti-CTLA4 naïve, they should receive the treatment concurrently.

The subgroup of patients without therapeutic intake of dexamethasone (= no intake or prophylactic intake) showed a significantly longer OS when being treated concurrently. In the literature, the use of dexamethasone is reported to be associated with an impairment of treatment outcome of ICI [47,48].

In the subgroup of patients with >4 mg, the timing of RT-ICI application made no difference. We suggest that a therapeutic use of steroids impairs the effect of ICI in the concurrent setting. Therefore, especially if patients do not need a therapeutic dose of dexamethasone, RT-ICI should be administered concurrently.

To summarize the subgroup analysis, patients with advanced-stage cancer, especially without melanoma as the cancer type, low inflammatory status before treatment, low administrable RT dose, a higher BM number, and PTV without the need for a therapeutic dose of dexamethasone seem to benefit most from concurrent RT-ICI treatment. Especially in the first-line setting (anti-CTLA4 naïve), the concurrent application should be endeavored.

### 4.4. Distinctive Reactions of the Immune System to Concurrent RT-ICI

We further analyzed abscopal effects (AbEs) and pseudoprogression (PsP) in this patient cohort, as defined earlier. We had a lot of missing values for abscopal effects. Although AbEs were low in number, all of them occurred in the concurrent RT-ICI group.

This radiation-induced shrinkage of distant, non-treated lesions is considered as evidence for effective immune stimulation by RT [18,19,20]. Due to their rare occurrence in the pre-ICI area, AbEs might have been underestimated in clinical routine. Abscopal effect rates of 25–52% are reported in the current literature when combined treatment concepts with RT and ICIs are used [49,50,51]. The optimal RT dose range and timing of RT-ICI application to boost abscopal effects remain unclear to date. Patients in our cohort had normofractionated (*n* = 2) or SRS treatment (*n* = 3). Considering the fact that we only detected abscopal effects in concurrently treated patients (*n* = 5, 16.1%), we suggest that the application of both treatments within a short time (here, 1 month) favors the occurrence of abscopal responses.

We also observed PsP more frequently in the group with concurrent RT-ICI (*n* = 12, 22.6%). PsP is a known imaging finding resulting from ICI or RT and seems to occur more frequently when both treatments are combined [52,53]. The transient increase in contrast-enhancing lesions may be due to immune cells being attracted to the tumor by certain mechanisms such as the release of neoantigens by RT or inflammation related to ICI therapy [54]. It regresses spontaneously or at least stabilizes at follow-up without any change in treatment [22,55].

The presence of IrAEs had a significant benefit regarding OS after 12 months’ follow-up time, and occurred more frequently in the concurrently treated RT-ICI group.

Patients in our cohort presenting immune reactions such as AbEs, PsP, or IrAEs showed longer OS rates. These findings correlate with recent publications: Theelen et al. observed significantly increased responses and outcomes in a pooled analysis of the Pembro-RT and MDACC trials in NSLCL patients after RT of a metastasis in combination with PD-1 inhibitor and at least one untreated metastasis observed for abscopal effects. Patients in the combined RT-ICI group had significantly better abscopal response rates and significantly better median PFS and OS without additional safety concerns [56].

Prior studies reporting about PsP after ICI found significantly better outcomes in patients with PsP than without [57,58]. The favorable outcome of patients with PsP may be related to the transient enlargement of lesions by infiltration of inflammatory cells, which may in part be associated with the favorable effect of T-cell infiltration [59]. Overall, there are limited data on this topic, especially regarding RT-ICI treatment.

Published data suggest that objective responses rates appear to be superior in ICI-treated patients who develop IrAEs. In a recent cohort study with 319 stage IV MM patients treated with first-line PD-1-based ICI, the presence of any grade IrAEs was significantly associated with longer OS and a higher percentage of patients with IrAEs had disease control compared to those without IrAEs [60].

We conclude from these findings and our analyses that distinctive reactions of the immune system to RT-ICI occur more frequently when both treatments are applied concurrently and lead to longer OS rates. Treatment response may be delayed when treatments are applied concurrently. To distinguish actual progression from an immune reaction, repeated imaging is necessary and should be performed before treatment change.

### 4.5. Toxicity Analysis

The timing of the onset of AEs due to ICI is unpredictable. Side effects may also occur months after the last administration and thus may fall within the RT period in sequential therapy, which makes it difficult to differentiate combination-related IrAEs from IrAEs due to ICI alone. That is why we decided to not distinguish between acute and late IrAEs.

There were more IrAEs and more acute CNS toxicities in the concurrent RT-ICI group, but the difference was not statistically significant (see Table A5). AEs were mostly mild and we detected no toxicity-related deaths.

This is in line with the current literature: Sha et al. report in a systematic review that comparable grade 3–4 toxicity occurred when RT-ICI was applied compared with ICI alone. Stratification by timing of RT and irradiated site revealed no significant differences, with only anti-CTLA4 in MM showing increased toxicity [61].

In our analysis, radionecrosis was rare and occurred more often in the concurrently treated group (11.7% vs. 3.4%), but the difference was not statistically significant.

Literature regarding the increased risk of adverse radiation effects such as radionecrosis in patients treated concurrently with RT-ICI, especially SRS, is inconsistent, including meta-analyses and reviews indicating that the risk of adverse effects is not increased [39,62,63]. Overall, radionecrosis rates of 0–37% are reported after SRS. More often, these rates are increased when combined with anti-CTLA4 antibodies, again highlighting their increased toxicity [64,65]. The conflicting data of radionecrosis rates are certainly debatable, but the more precise the RT and the better the adherence to dose–volume constraints, the less radionecrosis will be observed.

Since the volume of the lesions is probably the greatest risk factor for occurrence of radionecrosis, local therapy should be applied early, when lesions are still small.

### 4.6. Limitations

Due to its retrospective character and heterogeneous patient collective, our analysis has statistical weaknesses. Patient numbers were not big enough to include more parameters of interest in the multivariate Cox regression analysis. More substantial statistical analyses of subgroups would also require a higher total number of patients. Nevertheless, our findings reveal trends for relevant parameters that need to be verified in larger studies.

## 5. Conclusions

The concurrent use of RT and anti-PD-1 inhibitors prolongs survival in patients with BM of any cancer type and limited prognostic status. In our study, independent prognostic markers for OS were ECOG, cancer type, PTV, and concurrent application of RT-ICI.

The concurrent use of RT proved to be a valuable partner for anti-PD-1 treatment in our real-world patient cohort, resulting in 17.61 months’ median OS after almost 2 years of follow-up time and 95.3% LC rate after 6 months with a mild toxicity profile. Treatment response was delayed when patients were concurrently treated, possibly due to immune reactions of the treated lesions. Specific immune responses, such as AbEs, PsP, or the presence of IrAEs, occurred more frequently when RT and ICI were used concurrently and resulted in longer OS rates. If an immune reaction is suspected, a change in treatment should not be precipitated, but the patient should be closely monitored to avoid overtreatment.

Based on subgroup analyses regarding the timing of RT-ICI application, early and concurrent treatment seems to be beneficial, especially in first-line settings, in patients with low inflammatory status and cancers other than melanoma, without therapeutic dexamethasone intake, and in more and larger BMs where ablative doses cannot be administered, to boost immunogenic effects and achieve better treatment outcomes.

Future trials should consider all these parameters for further steps toward prescribed RT-ICI concepts for more efficient long-term immune responses.

## Figures and Tables

**Table 1:** Patient, lesion, and treatment characteristics of the entire patient cohort.

Patient and Lesion Characteristics	All Patients (*n* = 93)
Gender (female)	
female	38 (40.9%)
male	55 (59.1%)
Age (years), mean ± STD	62.1 ± 13.2
ECOG-PS	
0	28 (31.5%)
1	41 (46.1%)
>1	20 (22.5%)
Cancer type	
MM	65 (70.7%)
NSCLC	21 (22.8%)
other	6 (6.5%)
NLR	
<3 (low)	35 (44.9%)
≥3 (high)	43 (55.1%)
LDH	
≤ULN (245 U/L)	38 (55.9%)
>ULN (245 U/L)	30 (44.1%)
Extracranial disease	41 (47.1%)
Number of BMs, mean ± STD	3.4 ± 3.4
Total PTV (cm^3^), mean ± STD	277.5 ± 452.9
Neurological symptoms	38 (43.2%)
Treatment characteristics	
RT concept	
SRS	65 (69.9%)
WBRT	21 (22.6%)
other	7 (7.5%)
BED (Gy), mean ± STD	55.7 ± 10.1
RT courses	
1	32 (35.2%)
>1	59 (64.8%)
RT timing	
concurrently	63 (67.7%)
before ICI	19 (20.4%)
after ICI	11 (11.8%)
Dexamethasone	
no	10 (11.6%)
prophylactic dose ≤4 mg	59 (68.6%)
therapeutic dose >4 mg	17 (19.8%)
ICI duration (weeks), mean ± STD	22.2 ± 22.8
Prior systemic treatment	61 (68.5%)
Prior anti-CTLA4 treatment	22 (24.2%)

STD = standard deviation; ECOG-PS = Eastern Cooperative Oncology Group Performance Status; MM = malignant melanoma; NSCLC = non-small cell lung carcinoma; RCC = renal cell carcinoma; NHL = non-Hodgkin lymphoma; NLR = neutrophil-to-lymphocyte ratio; LDH = lactate dehydrogenase; ULN = upper limit of normal; BM = brain metastasis; PTV = planning target volume; RT = radiotherapy; SRS = stereotactic radiosurgery; WBRT = whole brain radiation therapy; BED = biologically effective dose; ICI = immune checkpoint inhibitor; anti-CTLA4 = anticytotoxic T-lymphocyte-associated protein 4.

**Table 2:** Patient, lesion, and treatment characteristics with respect to concurrent and non-concurrent RT-ICI therapy.

Patient and Lesion Characteristics	Non-Concurrent RT-ICI (*n* = 30)	Concurrent RT-ICI (*n* = 63)	*p*-Value
Gender (female)	12 (40%)	26 (41.3%)	0.907
Age (years), mean ± STD	61 ± 12.7	62,7 ± 13.5	0.495
ECOG-PS			0.457
0	7 (24.1%)	21 (35%)	
1	16 (55.2%)	25 (41.7%)	
>1	6 (20.7%)	14 (23.3%)	
Cancer type			0.823
MM	20 (66.7%)	45 (72.6%)	
NSCLC	8 (26.7%)	13 (21%)	
other	2 (6.7%)	4 (6.5%)	
NLR			0.421
<3 (low)	10 (38.5%)	25 (48.1%)	
≥3 (high)	16 (61.5%)	27 (51.9%)	
LDH			0.006
≤ULN (245 U/L)	8 (33.3%)	30 (68.2%)	
>ULN (245 U/L)	16 (66.7%)	14 (31.8%)	
Extracranial disease	11 (37.9%)	30 (51.7%)	0.224
Number of BMs			0.145
≤2	21 (72.4%)	35 (56.5%)	
>2	8 (27.6%)	27 (43.5%)	
Total PTV (cm^3^), mean ± STD	192.1 ± 388.5	318.7 ± 478.6	0.046
Total PTV (cm^3^)			0.170
≤3	15 (53.6%)	22 (37.9%)	
>3	13 (46.4%)	36 (62.1%)	
Neurological symptoms	10 (35.7%)	28 (46.7%)	0.334
Treatment characteristics			
RT concept			0.306
SRS	24 (80%)	41 (65.1%)	
WBRT	5 (16.7%)	16 (25.4%)	
other	1 (3.3%)	6 (9.5%)	
BED (Gy), mean ± STD	58 ± 11.1	54.6 ± 9.5	0.143
BED (Gy)			0.319
<60	8 (27.6%)	23 (38.3%)	
≥60	21 (72.4%)	37 (61.7%)	
RT courses			0.107
1	14 (46.7%)	18 (29.5%)	
>1	16 (53.3%)	43 (70.5%)	
RT timing			<0.001
concurrently	0 (0%)	63 (100%)	
before ICI	19 (63.3%)	0 (0%)	
after ICI	11 (36.7%)	0 (0%)	
Dexamethasone			0.055
no	1 (3.6%)	9 (15.5%)	
prophylactic dose ≤4 mg	24 (85.7%)	35 (60.3%)	
therapeutic dose >4 mg	3 (10.7%)	14 (24.1%)	
ICI duration (weeks), mean ± STD	15.9 ± 19.2	27.0 ± 24.3	0.067
Prior systemic treatment	23 (79.3%)	38 (63.3%)	0.128
Prior anti-CTLA4 treatment	11 (36.7%)	11 (18%)	0.051

STD = standard deviation; ECOG-PS = Eastern Cooperative Oncology Group Performance Status; MM = malignant melanoma; NSCLC = non-small cell lung carcinoma; RCC = renal cell carcinoma; NHL = non-Hodgkin lymphoma; NLR = neutrophil-to-lymphocyte ratio; LDH = lactate dehydrogenase; ULN = upper limit of normal; BM = brain metastasis; PTV = planning target volume; RT = radiotherapy; SRS = stereotactic radiosurgery; WBRT = whole brain radiation therapy; BED = biologically effective dose; ICI = immune checkpoint inhibitor; anti-CTLA4 = anticytotoxic T-lymphocyte-associated protein 4.

**Table 3:** Follow-up and outcome data of the entire RT-ICI cohort.

Follow-Up/Outcome	All Patients (*n* = 93)
Follow-up (months), mean ± STD	23.8 ± 24.3
OS (months), median (95% CI)	12.19 (4.36–20.02)
OS status	
alive	23 (25.8%)
dead	66 (74.2%)
PFS (months), median (95% CI)	4.70 (2.53–6.86)
LC	
3 months	52 (69.3%)
6 months	50 (89.3%)
Overall response rate	
CR	7 (7.6%)
PR	15 (16.3%)
SD	15 (16.3%)
PD	55 (59.8%)
Clinical benefit (CR + PR + SD)	37 (40.2%)
Progression rate	
cerebral progression	13 (14.3%)
systemic progression	13 (14.3%)
overall progression	39 (42.9%)
no progression	26 (28.6%)
cerebral response rate	39 (42.9%)
abscopal effects	5 (9.1%)
pseudoprogression	13 (17.8%)

STD = standard deviation; CI = confidence interval; OS = overall survival; RT = radiotherapy; ICI = immune checkpoint inhibitor; PFS = progression-free survival; LC = local control; CR = complete remission; PR = partial remission; SD = stable disease; PD = progressive disease; overall progression = cerebral and systemic progression.

**Table 4:** Univariate Cox proportional hazard regression analysis for OS and PFS.

Characteristics for Univariate Cox Regression Analysis	OS HR (95% CI)	*p*-Value	PFS HR (95% CI)	*p*-Value
Gender (reference: male)				
female vs. male	1.256 (0.765–2.062)	0.368	1.267 (0.798–2.011)	0.316
Age (years) (reference: ≤65)				
>65 vs. ≤65 years	1.149 (0.701–1.883)	0.581	0.83 (0.523–1.317)	0.429
ECOG-PS (reference: ECOG 0); overall log rank		0.031		0.255
1 vs. 0	1.823 (0.991–3.354)	0.053	1.523 (0.896–2.590)	0.120
≥2 vs. 0	2.532 (1.228–5.222)	0.012	1.515 (0.784–2.929)	0.217
Cancer type (reference: other)				
MM vs. other	0.457 (0.267–0.782)	0.004	0.628 (0.382–1.033)	0.067
NLR (reference: low (<3))				
≥3 (high) vs. <3 (low)	2.037 (1.184–3.506)	0.010	1.318 (0.81–2.143)	0.266
LDH (reference: normal, ≤ULN)				
>ULN (245 U/L) vs. ≤ULN (245 U/L)	1.853 (1.059–3.241)	0.031	1.066 (0.630–1.803)	0.812
Extracranial disease (reference: no)				
yes vs. no	1.132 (0.684–1.875)	0.629	0.767 (0.484–1.214)	0.257
Number of BM (reference: ≤2 BM)				
>2 vs. ≤2	1.154 (0.691–1.926)	0.585	1.616 (1.004–2.599)	0.048
PTV (cm^3^) (reference: ≤3)				
>3 vs. ≤3	2.213 (1.305–3.754)	0.003	1.819 (1.124–2.943)	0.015
Neurological symptoms (reference: no)				
yes vs. no	2.114 (1.285–3.478)	0.003	1.424 (0.897–2.262)	0.134
RT concept (reference: SRS)				
WBRT vs. SRS	1.985 (1.112–3.543)	0.019	1.828 (1.053–3.174)	0.032
BED (Gy) (reference: <60)				
≥60 vs. <60	0.519 (0.309–0.871)	0.013	0.599 (0.370–0.969)	0.037
RT courses (reference: 1)				
>1 vs. 1	0.725 (0.433–1.213)	0.221	0.620 (0.386–0.995)	0.048
RT timing, 12 months (reference: non-concurrent)				
concurrent vs. non-concurrent	0.527 (2.86–0.973)	0.041	0.883 (0.526–1.485)	0.640
RT timing (reference: concurrent); overall log rank		<0.001		0.067
RT after ICI vs. concurrent	3.971 (1.839–7.814)	<0.001	2.104 (1.079–4.099)	0.029
RT before ICI vs. concurrent	0.955 (0.504–1.809)	0.887	0.964 (0.546–1.703)	0.900
Dexamethasone application >4 mg (reference: no)				
yes vs. no	1.698 (0.941–3.063)	0.079	1.477 (0.833–2.617)	0.182
Prior systemic treatment (reference: no)				
yes vs. no	0.983 (0.581–1.663)	0.950	1.194 (0.724–1.968)	0.488
Prior anti-CTLA4 treatment (reference: no)				
yes vs. no	0.498 (0.271–0.914)	0.024	0.586 (0.338–1.014)	0.056
Abscopal effects (reference: no)				
yes vs. no	0.847 (0.301–2.379)	0.752	2.036 (0.786–5.271)	0.143
Pseudoprogression (reference: no)				
yes vs. no	0.687 (0.32–1.474)	0.335	0.646 (0.316–1.319)	0.230
Immune-related adverse events (reference: no)				
yes vs. no	0.677 (0.399–1.15)	0.149	0.637 (0.388–1.046)	0.075
Radionecrosis (reference: no)				
yes vs. no	1.152 (0.523–2.54)	0.725	0.795 (0.364–1.734)	0.564

When comparing more than two categories, the *p*-value of the overall log rank test is also provided. OS = overall survival; PFS = progression-free survival; ECOG-PS = Eastern Cooperative Oncology Group Performance Status; MM = malignant melanoma; NLR = neutrophil-to-lymphocyte ratio; LDH = lactate dehydrogenase; ULN = upper limit of normal; BM = brain metastasis; PTV = planning target volume; RT = radiotherapy; SRS = stereotactic radiosurgery; WBRT = whole brain radiation therapy; BED = biologically effective dose; ICI = immune checkpoint inhibitor; anti-CTLA4 = anticytotoxic T-lymphocyte-associated protein 4.

**Table 5:** Multivariate Cox proportional hazard regression analysis for OS.

Characteristics for Multivariate Cox Regression Analysis	OS HR (95% CI)	*p*-Value
ECOG-PS (reference: ECOG 0)		
1 vs. 0	1.694 (0.873–3.289)	0.119
≥2 vs. 0	2.756 (1.253–6.061)	0.012
Cancer type (reference: other)		
MM vs. other	0.516 (0.288–0.926)	0.026
RT timing (reference: non-concurrent)		
concurrent vs. non-concurrent	0.539 (0.299–0.971)	0.040
RT concept (reference: SRS)		
WBRT vs. SRS	0.763 (0.280–2.077)	0.596
other vs. SRS	0.117 (0.014–1.006)	0.051
BED (Gy) (reference: <60)		
≥60 vs. <60	0.494 (0.195–1.252)	0.137
PTV (cm^3^) (reference: ≤3)		
>3 vs. ≤3	1.947 (1.007–3.763)	0.048

OS = overall survival; HR = hazard ratio; CI = confidence interval; ECOG-PS = Eastern Cooperative Oncology Group Performance Status; MM = malignant melanoma; RT = radiotherapy; SRS = stereotactic radiosurgery; WBRT = whole brain radiation therapy; BED = biologically effective dose; PTV = planning target volume.

**Table 6:** Follow-up and outcome data with respect to concurrent and non-concurrent RT-ICI therapy.

Follow-Up/Outcome	Non-Concurrent RT-ICI (*n* = 30)	Concurrent RT-ICI (*n* = 63)	*p*-Value
Follow-up (months), mean ± STD	25.7 ± 34.5	22.9 ± 17.6	0.591
OS (months), median (95% CI)	6.83 (2.15–11.52)	17.61 (6.02–29.20)	0.173
RT before ICI	10.15 (0.00–33.48)	-	
RT after ICI	2.20 (0.00–5.25)	-	
OS status			0.071
alive	4 (13.8%)	19 (31.7%)	
dead	25 (86.2%)	41 (68.3%)	
PFS (months), median (95% CI)	4.70 (1.18–7.01)	5.49 (1.80–9.18)	0.383
RT before ICI	5.29 (3.79–6.79)	-	
RT after ICI	2.14 (1.43–2.84)	-	
LC			
3 months	18 (81.8%)	34 (64.2%)	0.131
6 months	9 (69.2%)	41 (95.3%)	0.008
Lesion response at 3 months			0.552
smaller/stable	17 (65.4%)	30 (51.7%)	
larger/new bm	3 (11.5%)	11 (19%)	
mixed response	1 (3.8%)	8 (13.8%)	
Lesion response at 6 months			0.107
smaller/stable	8 (61.5%)	37 (75.5%)	
larger/new bm	2 (15.4%)	0 (0%)	
mixed response	1 (7.7%)	2 (4.1%)	
Overall response rate			0.151
CR	1 (3.3%)	6 (9.7%)	
PR	7 (23.3%)	8 (12.9%)	
SD	2 (6.7%)	13 (21%)	
PD	20 (66.7%)	35 (56.5%)	
Clinical benefit (CR + PR + SD)	10 (33.3%)	27 (43.5%)	
Progression rate			0.015
cerebral progression	3 (10%)	10 (16.4%)	
systemic progression	7 (23.3%)	6 (9.8%)	
overall progression	17 (56.7%)	22 (36.1%)	
no progression	3 (10%)	23 (37.7%)	
cerebral response rate	10 (33.3%)	29 (47.5%)	0.198
abscopal effects	0 (0%)	5 (16.1%)	0.039
pseudoprogression	1 (5%)	12 (22.6%)	0.079

STD = standard deviation; CI = confidence interval; OS = overall survival; RT = radiotherapy; ICI = immune checkpoint inhibitor; PFS = progression-free survival; LC = local control; CR = complete remission; PR = partial remission; SD = stable disease; PD = progressive disease; overall progression = cerebral and systemic progression.

## Data Availability

The data are included in the article. Further details can be obtained from the corresponding author.

## Appendix A

**Table A1:** Patient, lesion, and treatment characteristics.

Patient and Lesion Characteristics	All Patients (*n* = 93)	Non-Concurrent RT-ICI (*n* = 30)	Concurrent RT-ICI (*n* = 63)	*p*-Value
BMI (kg/m^2^)				0.469
UW (<18.5)	4 (4.8%)	0 (0%)	4 (7.3%)	
NW (18.5–24.9)	27 (32.1%)	10 (34.5%)	17 (30.9%)	
OW (25.0–29.9)	28 (33.3%)	11 (37.9%)	17 (30.9%)	
OB (≥30)	25 (29.8%)	8 (27.6%)	17 (30.9%)	
PD-L1 status				0.640
≥1% (positive)	20 (51.3%)	4 (44.4%)	16 (53.3%)	
<1% (negative)	19 (48.7%)	5 (55.6%)	14 (46.7%)	
High tumor burden	55 (62.5%)	15 (51.7%)	40 (67.8%)	0.143
Treatment characteristics				
Prior RT	41 (45.6%)	13 (43.3%)	28 (46.7%)	0.765
ICI type				0.426
pembrolizumab	63 (67.7%)	22 (73.3%)	41 (65.1%)	
nivolumab	30 (32.3%)	8 (26.7%)	22 (34.9%)	
ICI duration (weeks), mean ± STD	22.2 ± 22.8	15.9 ± 19.2	27.0 ± 24.3	0.067
Prior systemic treatment	61 (68.5%)	23 (79.3%)	38 (63.3%)	0.128

BMI = body mass index; OW = overweight; UW = underweight; NW = normal weight; OB = obese; PD-L1 = programmed death-ligand 1; RT = radiotherapy; ICI = immune checkpoint inhibitor. High tumor burden: Defined as multiple metastases (*n* > 10) intra- and/or extracranial.

**Table A2:** Characteristics for univariate Cox regression analysis.

Characteristics for Univariate Cox Regression Analysis	OS HR (95% CI)	*p*-Value	PFS HR (95% CI)	*p*-Value
BMI (kg/m^2^) (reference: OW); overall log rank		0.127		<0.001
UW vs. OW	3.354 (0.982–11.460)	0.054	12.471 (3.415–45.542)	<0.001
NW vs. OW	1.072 (0.561–2.046)	0.834	1.358 (0.753–2.446)	0.309
OB vs. OW	1.590 (0.854–2.961)	0.144	1.590 (0.877–2.885)	0.127
PD-L1 status (reference: positive)				
negative vs. positive	0.678 (0.293–1.57)	0.364	1.421 (0.683–2.958)	0.348
High tumor burden (reference: no)				
yes vs. no	1.223 (0.733–2.042)	0.440	0.957 (0.6–1.527)	0.854
ICI type (reference: pembrolizumab)				
nivolumab vs. pembrolizumab	0.994 (0.575–1.719)	0.982	1.282 (0.782–2.1)	0.325
Prior systemic treatment (reference: no)				
yes vs. no	0.983 (0.581–1.663)	0.950	1.194 (0.724–1.968)	0.488

BMI = body mass index; OW = overweight; UW = underweight; NW = normal weight; OB = obese; PD-L1 = programmed death ligand 1; ICI = immune checkpoint inhibitor. High tumor burden: Defined as multiple metastases (*n* > 10) intra- and/or extracranial.

**Table A3:** Patient, lesion, and treatment characteristics regarding MM vs. other cancer types.

Patient and Lesion Characteristics	All Patients (*n* = 92)	Other Cancer Types (*n* = 27)	Malignant Melanoma (*n* = 65)	*p*-Value
Gender (female)	38 (41.3%)	7 (25.9%)	31 (47.7%)	0.054
Age (years), mean ± STD	62.1 ± 13.2	58.5 ± 9.1	63.6 ± 14.4	0.049
ECOG-PS				0.049
0	28 (31.5%)	8 (30.8%)	20 (31.7%)	
1	41 (46.1%)	8 (30.8%)	33 (52.4%)	
>1	20 (22.5%)	10 (38.5%)	10 (15.9%)	
NLR				0.180
<3 (low)	35 (44.9%)	6 (31.6%)	29 (49.2%)	
≥3 (high)	43 (55.1%)	13 (68.4%)	30 (50.8%)	
LDH				0.651
≤ULN (245 U/L)	38 (55.9%)	6 (50.0%)	32 (57.1%)	
>ULN (245 U/L)	30 (44.1%)	6 (50.0%)	24 (42.9%)	
Extracranial disease	41 (47.1%)	14 (53.8%)	27 (44.3%)	0.412
Number of BMs				0.272
≤2	55 (61.1%)	13 (52%)	42 (64.6%)	
>2	35 (38.9%)	12 (48%)	23 (35.4%)	
Total PTV (cm^3^)				0.036
≤3	37 (43.0%)	6 (25%)	31 (50%)	
>3	49 (57.0%)	18 (75%)	31 (50%)	
Neurological symptoms	38 (43.2%)	14 (56.0%)	24 (38.1%)	0.126
Treatment characteristics				
RT concept				0.057
SRS	64 (69.6%)	14 (51.9%)	50 (76.9%)	
WBRT	21 (22.8%)	10 (37.0%)	11 (16.9%)	
other	7 (7.6%)	3 (11.1%)	4 (6.2%)	
BED (Gy)				0.004
<60	31 (34.8%)	15 (57.7%)	16 (25.4%)	
≥60	58 (65.2%)	11 (42.3%)	47 (74.6%)	
RT courses				0.808
1	32 (35.2%)	10 (37.0%)	22 (34.4%)	
>1	59 (64.8%)	17 (63.0%)	42 (65.6%)	
RT timing				0.811
concurrently	62 (67.4%)	17 (63.0%)	45 (69.2%)	
before ICI	19 (20.7%)	6 (22.2%)	13 (20.0%)	
after ICI	11 (12.0%)	4 (14.8%)	7 (10.8%)	
Dexamethasone				0.097
no	10 (11.6%)	4 (16.0%)	6 (9.8%)	
prophylactical ≤4 mg	59 (68.6%)	13 (52.0%)	46 (75.4%)	
therapeutical >4 mg	17 (19.8%)	8 (32.0%)	9 (14.8%)	
ICI duration (weeks), mean ± STD	22.2 ± 22.8	19.7 ± 23.3	22.9 ± 22.8	0.352
Prior systemic treatment	61 (68.5%)	25 (96.2%)	36 (57.1%)	<0.001
Prior anti-CTLA4	22 (24.4%)	0 (0%)	22 (34.9%)	<0.001

MM = malignant melanoma; ECOG-PS = Eastern Cooperative Oncology Group Performance Status; MM = malignant melanoma; NLR = neutrophil-to-lymphocyte ratio; LDH = lactate dehydrogenase; ULN = upper limit of normal; BM = brain metastasis; PTV = planning target volume; RT = radiotherapy; SRS = stereotactic radiosurgery; WBRT = whole brain radiation therapy; BED = biologically effective dose; ICI = immune checkpoint inhibitor; anti-CTLA4 = anticytotoxic T-lymphocyte-associated protein 4.

**Table A4:** Selected OS rates for 6, 12, 24, and 36 months regarding different covariates.

Characteristics for Selected OS Rates	6-Month OS Rate	12-Month OS Rate	24-Month OS Rate	36-Month OS Rate	*p*-Value (Log Rank)
All patients (*n* = 93)	70.8%	50.7%	40.0%	32.1%	
ECOG-PS					0.031
0	88.7%	69.4%	61.3%	52.4%	
1	68.3%	48.8%	36.6%	26.6%	
>1	50.0%	27.8%	16.7%	0%	
Cancer type					0.003
MM	77.8%	57.1%	44.3%	37.6%	
other	51.8%	33.0%	28.3%	17.7%	
NLR					0.009
<3 (low)	85.3%	61.8%	55.9%	46.6%	
≥3 (high)	58.5%	39.0%	26.8%	20.1%	
LDH					0.028
≤ULN (245 U/L)	89.5%	68.4%	47.4%	39.5%	
>ULN (245 U/L)	50.0%	30.0%	30.0%	26.3%	
PTV					0.002
≤3 cm^3^	89.2%	62.2%	53.8%	44.8%	
>3 cm^3^	53.9%	38.1%	26.9%	22.4%	
Neurological symptoms					0.003
yes	50.0%	34.2%	26.3%	21.1%	
no	87.0%	65.3%	52.1%	42.5%	
RT concept					0.016
SRS	80.2%	55.1%	43.3%	34.2%	
WBRT	36.8%	26.3%	21.1%	21.1%	
BED					0.011
<60	41.4%	31.0%	24.1%	24.1%	
≥60	85.8%	60.8%	48.2%	35.7%	
RT-ICI application					0.173
concurrently	77.4%	58.1%	44.0%	34.2%	
non-concurrently	57.1%	35.7%	32.1%	28.1%	
RT timing					<0.001
concurrently	77.4%	58.1%	44.0%	34.2%	
before ICI	76.5%	47.1%	47.1%	41.2%	
after ICI	27.3%	18.2%	9.1%	9.1%	
Dexamethasone application					0.075
>4 mg	41.2%	29.4%	29.4%	23.5%	
≤4 mg	77.1%	55.5%	43.1%	34.8%	
Prior anti-CTLA4 treatment					0.022
yes	90.9%	68.2%	50.0%	50.0%	
no	63.8%	44.7%	36.6%	25.3%	
Abscopal effects					0.752
yes	100%	80.0%	40.0%	40.0%	
no	64.0%	46.0%	34.0%	26.0%	
Pseudoprogression					0.332
yes	100%	76.9%	53.8%	46.2%	
no	78.0%	53.8%	42.6%	32.0%	
Immune-related adverse events					0.147
yes	79.1%	62.8%	46.2%	35.6%	
no	62.5%	37.5%	31.3%	28.1%	

OS = overall survival; ECOG-PS = Eastern Cooperative Oncology Group Performance Status; MM = malignant melanoma; NLR = neutrophil-to-lymphocyte ratio; LDH = lactate dehydrogenase; ULN = upper limit of normal; PTV = planning target volume; RT = radiotherapy; SRS = stereotactic radiosurgery; WBRT = whole brain radiation therapy; BED = biologically effective dose; ICI = immune checkpoint inhibitor; anti-CTLA4 = anticytotoxic T-lymphocyte-associated protein 4.

**Table A5:** Adverse events (AEs) and CTCAE grades of all patients and the two treatment groups with non-concurrent and concurrent RT-ICI therapy.

Adverse Events (AEs)	All Patients (*n* = 93)	Non-Concurrent RT-ICI (*n* = 30)	Concurrent RT-ICI (*n* = 63)	*p*-Value
All AEs	60 (74.1%)	19 (70.4%)	41 (75.9%)	0.591
CTCAE Grade 1	10 (16.7%)	3 (15.8%)	7 (17.1%)	0.746
CTCAE Grade 2	33 (55%)	9 (47.4%)	24 (58.5%)	
CTCAE Grade 3	12 (20%)	5 (26.3%)	7 (17.1%)	
CTCAE Grade 4	1 (1.7%)	0 (0%)	1 (2.4%)	
Immune-related AEs	44 (56.4%)	12 (46.2%)	32 (61.5%)	0.196
CTCAE Grade 1	11 (25%)	3 (25%)	8 (25%)	0.071
CTCAE Grade 2	24 (54.5%)	4 (33.3%)	20 (62.5%)	
CTCAE Grade 3	6 (13.6)	3 (25%)	3 (9.4%)	
CTCAE Grade 4	1 (2.3%)	0 (0%)	1 (3.1%)	
Acute CNS toxicity	27 (35.1%)	6 (22.2%)	21 (42%)	0.083
CTCAE Grade 1	4 (14.8%)	1 (16.7%)	3 (14.3%)	0.884
CTCAE Grade 2	17 (63%)	3 (50%)	14 (66.7%)	
CTCAE Grade 3	3 (11.1%)	1 (16.7%)	2 (9.5%)	
Fatigue	25 (32.1%)	8 (29.6%)	17 (33.3%)	0.739
CTCAE Grade 1	10 (40%)	1 (12.5%)	9 (52.9%)	0.104
CTCAE Grade 2	9 (36%)	3 (37.5%)	6 (3.3%)	
CTCAE Grade 3	5 (20%)	3 (37.5%)	2 (11.8%)	
Other AEs	10 (13%)	4 (14.8%)	6 (12%)	0.726
CTCAE Grade 1	4 (40%)	1 (25%)	3 (50%)	0.251
CTCAE Grade 2	2 (20%)	0 (0%)	2 (33.3%)	
CTCAE Grade 3	3 (30%)	2 (50%)	1 (16.7%)	
Radionecrosis	8 (9%)	1 (3.4%)	7 (11.7%)	0.204

AEs = adverse events; CTCAE = Common Terminology Criteria for Adverse Events.
